# Ultrastructural analysis of human uncinate fasciculus with coherent anti-Stokes Raman spectroscopy

**DOI:** 10.1117/1.BIOS.3.2.025002

**Published:** 2026-03-17

**Authors:** Kelly Perlman, Valérie Pineau Noël, Armand Collin, Justine Major, Murielle Mardenli, Sébastien Jerczynski, Maria Antonietta Davoli, Julien Cohen-Adad, Daniel Côté, Naguib Mechawar

**Affiliations:** a Douglas Mental Health University Institute, Verdun, Canada; b McGill University, Integrated Program in Neuroscience, Montreal, Québec, Canada; c CERVO brain research center, Université, Department of Physics, Laval, Québec, Canada; d NeuroPoly Lab, Department of Electrical Engineering, Polytechnique Montreal, Montreal, Québec, Canada; e Mila - Quebec Artificial Intelligence Institute, Montreal, Québec, Canada; f McGill University, Department of Psychiatry, Montreal, Québec, Canada

**Keywords:** coherent anti-Stokes Raman spectroscopy, spectral-focusing coherent anti-Stokes Raman spectroscopy, deep learning, segmentation, myelin, postmortem, human brain

## Abstract

**Significance:**

Characterizing the ultrastructure of myelin in the human brain is key to understanding the neurobiology of both health and disease. In postmortem human brain tissue, electron microscopy is often technically unfeasible due to poorer tissue quality.

**Aim:**

The uncinate fasciculus (UF) is a long-range white matter association tract that connects the anterior temporal lobe with the orbitofrontal cortex. The UF is not present in rodents yet is highly expanded in humans and nonhuman primates. As such, its molecular and ultrastructural properties are virtually unknown.

**Approach:**

Here, we develop and validate a spectral-focusing coherent anti-Stokes Raman spectroscopy (sf-CARS) system coupled with a custom AxonDeepSeg segmentation model to characterize UF ultrastructure in the human postmortem brain (n=6).

**Results:**

We provide a proof of concept of this new methodological pipeline in the UF temporal segment and observe that the mean axon diameter detected is 0.93  μm±0.54 and the mean myelin thickness is 0.48  μm±0.14. We also observe that the UF axons are thicker than those in the anterior cingulate cortex white matter.

**Conclusions:**

We detail and validate the full methodology, including tissue fixation and sectioning, sf-CARS acquisition settings, as well as the AxonDeepSeg deep learning model parameters, such that this pipeline can be utilized by others in the field.

Statement of Discovery This work describes and validates a novel pipeline of spectral-focusing coherent anti-Stokes Raman spectroscopy (sf-CARS) coupled with a deep learning image segmentation model, which allows for robust measurement of postmortem white matter ultrastructure. We provide the first estimates of human uncinate fasciculus axon diameter, myelin thickness, and g-ratio.

## 1 Introduction

The uncinate fasciculus (UF) is a hook-shaped association white matter fiber tract that bidirectionally connects the anterior temporal lobe to the medial and lateral orbitofrontal cortex and part of the inferior frontal gyrus.^1^^,^^2^ This tract is sometimes referred to in segments, namely, the temporal segment, the insular segment, and the frontal segment.^2^^,^^3^ This tract is not present in rodents (not to be confused with the uncinate fascicle of the rodent cerebellum^4^^,^^5^) and has been studied mostly in humans and nonhuman primates via diffusion tensor imaging or gross dissection studies, and as such, it is virtually uncharacterized from molecular and ultrastructural perspectives.^2^ The closest information available is from a study that used electron microscopy to look at the point of intersection between the UF and the inferior occipitofrontal fasciculus in three human brains donated for medical education, with 4% formaldehyde fixation via infusion through the femoral artery less than 48 h postmortem.^6^ Electron microscopy in postmortem human brains is possible provided that preservation conditions are excellent^7^ such as those in rapid autopsy prograyelin sheathms; however, much of the postmortem human tissue available has longer postmortem intervals (PMIs) and is in poorer condition. As such, electron microscopy is unfeasible for most collected postmortem brain tissue. This is especially the case for brain banks with a specialized focus such as suicide, in which the brains typically have longer PMIs and refrigeration delays (time between death and storage of the body at 4°C), such as those of the Douglas Bell-Canada Brain Bank.^8^

Coherent anti-Stokes Raman scattering (CARS) microscopy presents an alternative method to obtain ultrastructural metrics, with a high sensitivity, label-free technique.^9^^–^^13^ This method capitalizes on the strong Raman scattering properties of lipids, by probing the methylene (${\mathrm{CH}}_{2}$) stretching vibrational band ($2845\;{\mathrm{cm}}^{-1}$).^14^^,^^15^ Fatty acid tails are rich in ${\mathrm{CH}}_{2}$ groups, and as myelin is extremely lipid rich (∼70% to 85% lipids by dry weight^16^), using the ${\mathrm{CH}}_{2}$ vibrational signature essentially creates a molecular map of myelin in a tissue slice.

Previously, our group utilized CARS microscopy to study the postmortem anterior cingulate cortex (ACC) in the context of depression and childhood abuse (CA).^15^ We observed alterations in gene expression and DNA methylation patterns related to myelination as well as ultrastructure changes exclusively in depressed individuals who died by way of suicide with a history of severe CA.^15^ Specifically, we found that, in small caliber axons (0.5 to 1.25  μm diameter) of the ACC white matter, individuals with a history of CA had thinner myelin and larger g-ratios than the comparator groups.^15^

As the publication of these ACC results, a newer version of CARS microscopy has been developed. Spectral-focusing CARS (sf-CARS) represents a significant advancement in label-free neurophotonics imaging, overcoming key limitations of traditional CARS systems that rely on picosecond pulses. By employing ultra-short femtosecond pulses across larger wavebands, sf-CARS utilizes temporal chirping through a glass rod to enhance spectral resolution according to the dispersion levels of the pump and the Stokes beams.^17^ This approach enables precise targeting of different Raman bands through controlled temporal overlap between pulses, facilitating fast hyperspectral imaging via automated delay stage manipulation and increasing image contrast by targeting specific Raman bands. Although the absorption cross-section of myelin is not naturally optimized for femtosecond pulse interactions, the chirping process creates a linear dispersion over time of frequencies within the envelope of the pulse, and with it, a reduced effective power per spectral component. This approach still enables successful myelin targeting despite the power-dependent nature of Raman scattering.

Furthermore, since the publication of our ACC results, segmentation approaches have been augmented with the implementation of deep learning. Although manual quantification remains the gold standard for morphometric assessment,^18^ this approach is labor-intensive, requiring extensive expert annotation time and specialized histological expertise. Recent advances in deep learning have introduced more efficient alternatives, particularly the U-Net architecture,^19^ which has demonstrated superior time efficiency and practicality compared with traditional manual methods for biomedical image segmentation. AxonDeepSeg represents a state-of-the-art segmentation framework that employs a U-Net-based architecture to perform multiclass semantic segmentation of axons and myelin sheaths while simultaneously extracting comprehensive morphometric parameters.^20^ This approach based on convolutional neural networks offers several advantages over manual quantification: reduced annotation burden for large-scale datasets, decreased processing time, and automated extraction of key morphometric features. The framework can effectively leverage manually annotated training data to generate accurate predictions on custom datasets across diverse microscopy modalities.

Therefore, with the advances in both microscopy and image segmentation technologies, we provide an updated, modernized workflow to quantify white matter ultrastructure in the postmortem human brain. Here, we validate this new method on the UF, providing both a proof of concept and the first-ever ultrastructural metrics of the UF temporal segment.

## 2 Methods

### 2.1 Tissue Collection

This research was approved by the Douglas Hospital Research Ethics Board. Frozen UF was dissected from the left Brodmann Area 38 (temporal pole). It was dissected from this region because the WM tract is purely UF and not contaminated with adjacent fibers such as the inferior frontal occipital fasciculus. The brains were obtained through the Douglas-Bell Canada Brain Bank^21^ thanks to a close collaboration with the Québec coroner’s office. Informed consent was obtained from the donors’ next of kin, and donor phenotypic information was acquired by way of a standardized psychological autopsy procedure. In this proof-of-concept study, we excluded subjects with psychiatric disorders and histories of childhood abuse and focused on “control” subjects only. Neurodegenerative disorders also constituted an exclusion criterion.

### 2.2 Tissue Fixation and Sectioning

**Fig. 1:**
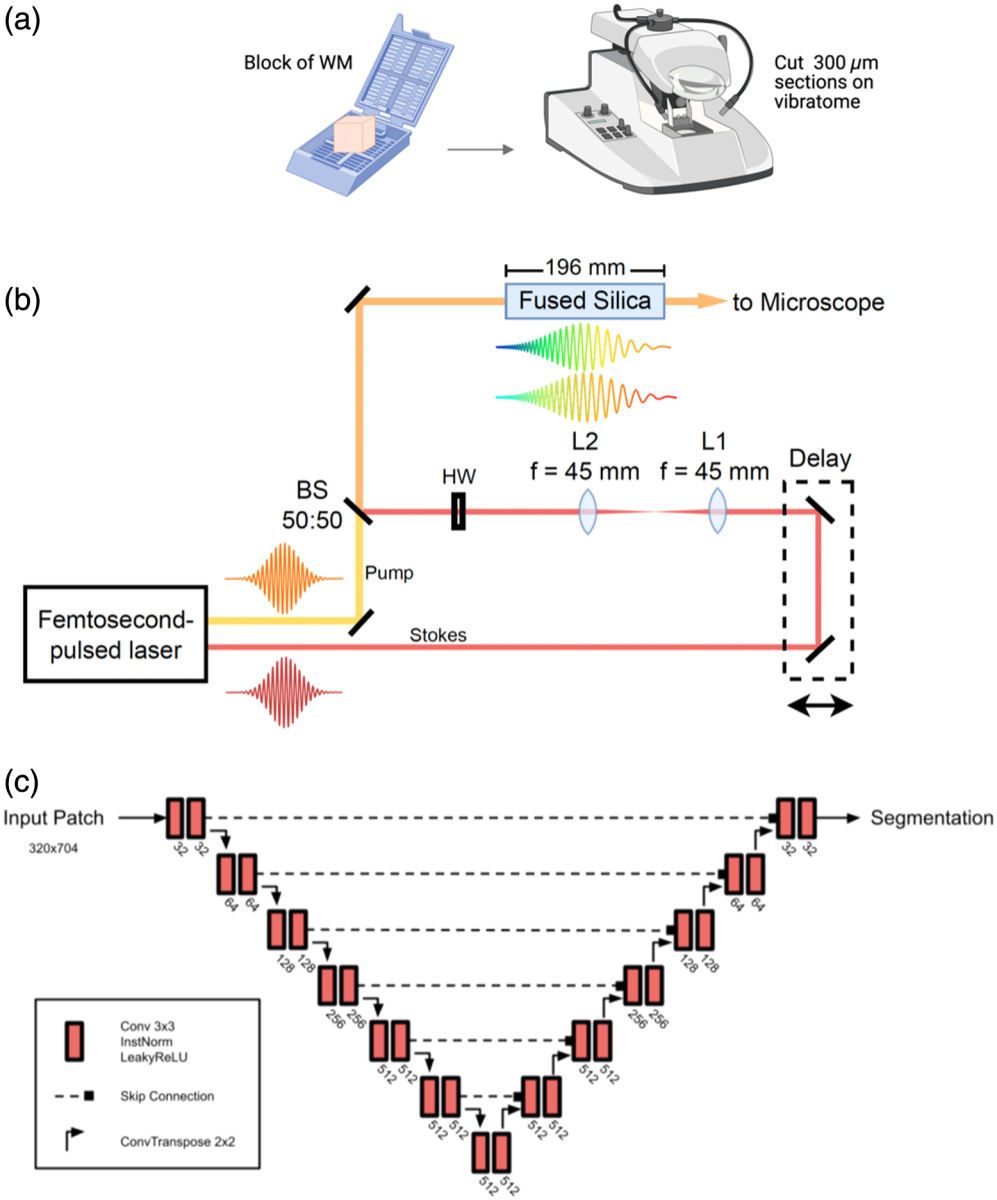
Summarized methods schematic. (a) A block of white matter ($∼1\;{\mathrm{cm}}^{3}$) is cut into 300  μm thick slices on a Leica Vibratome. (b) sf-CARS acquisition setup. Short 100 fs pulses from a ytterbium (Yb) laser pumping an oscillator are emitted with a defined polarization, frequency, and power. Fixed-wavelength laser beam of 1045 nm (Stokes) is temporally delayed by a linear motion stage. It is then re-collimated through a 4f-system to match the collimation and size of the tunable (Pump) beam of 805 nm and then rotated with a half-wave plate (HW) to match the polarization of the pump beam. Both beams are split by a beamsplitter (BS) and subsequently synchronized and combined. They are then passed through a fused silica glass rod for a negative chirping effect or sliding of frequencies. Pulses are elongated, which improves the spectral resolution. The light is then sent to the microscope. (c) Network architecture of the axon and myelin segmentation model. Each block contains convolution layers with 3×3 kernels, instance normalization, and LeakyReLU. Skip connections merge the encoder outputs with the decoder inputs at each stage. The decoder applies 2D transposed convolutions to upsample the feature maps in between stages.

Blocks of fresh frozen UF ($∼1\;{\mathrm{cm}}^{3}$) were fixed overnight in 10% formalin at 4°C and then stored in 1x PBS. The fixed tissue was then cut on a Leica VT1200S vibratome filled with ice-cold 1x PBS at 300  μm thick sections (settings: −0.8  mm amplitude, 0.8  mm/s speed, 300  μm step size) [Fig. 1(a)]. The slices were stored in vials with PBS with cryopreservative (glycerol and ethylene glycol) at −20°C. As the original orientation of the tissue at dissection was not preserved in the tissue cassettes, each block was split into three and sliced in all three orientations with respect to a preselected face of the cube. Upon imaging, the one section with the clear, abundant, dense fibers was selected as the correct orientation. The imaging was performed at the CERVO brain research center at Université Laval.

### 2.3 sf-CARS Microscopy

sf-CARS imaging was performed using a ytterbium (Yb) Insight X3 pulsed femtosecond (fs) laser system configured with two distinct output beams. The tunable laser was set to 805 nm with 100 fs pulses and ∼300  mW effective power at source output, whereas the fixed laser operated at 1045 nm with 100 fs pulses and ∼600  mW effective power at source output. These excitation wavelengths were selected to target the symmetric ${\mathrm{CH}}_{2}$ stretching frequency at $2845\;{\mathrm{cm}}^{-1}$. This frequency is a chemical signature for lipids, the primary constituent of the myelin sheath.^14^

Laser power settings were subsequently adjusted and optimized for each imaging session and sample type to achieve optimal contrast. Temporal chirping was achieved using a 19.6 cm fused silica rod with low dispersivity characteristics. Given the spectral widths of 9.0 and 7.3 nm (at 805 and 1045 nm, respectively) (Fig. S1 in the Supplementary Material), after passing through the fused silica rod with a dispersion coefficients of 103 and 31  ps/nm/km, the pump beam is estimated to be 2.44 times transform-limited (or 250 fs from 105 fs), whereas the Stokes beam remains largely unaffected at 1.03 times the transform limit (at 233 fs), which makes them matched in pulse widths. Images were acquired through an Olympus 60X/0.8 NA water immersion objective with 2× additional optical zoom utilizing a galvanometer and a polygonal mirror for scanning.^22^ The photomultiplier tube voltage was maintained at 750 V, and each final image represented an average of 100 frames to enhance the signal-to-noise ratio by reducing photon noise. Image acquisition (8 bits, maximum of 255) was performed over 500×1000  pixels at 30 frames per second. A tunable offset was used during the acquisition to eliminate low counts of photons and was adjusted for each image to optimize contrast and signal-to-noise ratio. Plus, both linear motion stages in the optical path (delay and at sample) were manually fine-tuned to achieve maximum contrast, and captured images underwent post-processing correction for enhanced contrast analysis. The sf-CARS acquisition setup is summarized in Fig. 1(b).

### 2.4 sf-CARS Validation

To evaluate the spectral resolution, Raman spectra of 10-μm polystyrene beads (Polybeads^®^ Microspheres 10.00  μm, Polysciences, Warrington, Pennsylvania, United States) were acquired. Polystyrene produces a strong Raman signal in the CH stretching vibrational region,^23^ which is also the region of interest to target the Raman signal from lipids in myelin. For these measurements, the photomultiplier tube was replaced by an optical fiber (M59L01, Thorlabs, Newton, New Jersey, United States) connected to a spectrometer (USB 2000+, OceanOptics, Orlando, Florida, United States). Ten spectra are acquired with and without the glass rod in the excitation path using an exposure time of 5000 and 1000 ms, respectively. After baseline correction and normalization of the summed spectra, the full-width half-maximum (FWHM) of the Raman signal between 2800 and $3100\;{\mathrm{cm}}^{-1}$ was calculated to quantify the rod’s impact on the spectral resolution. In addition, to evaluate the overlap between the pump and the Stokes beams, we measured the total CARS intensity of 10-μm polystyrene bead images. This assessment was performed both with and without the presence of the rod while systematically adjusting the temporal delay.

### 2.5 Deep-Learning-Based Morphometry Analysis

Deep-learning-based axon segmentation typically comprises four sequential stages: data preparation, model training, result evaluation, and inference. During data preparation, microscopy images with corresponding axon and myelin annotations are normalized to consistent resolution parameters, partitioned into smaller image patches, and intensity is rescaled to the [0, 1] range. The model training phase utilizes manually segmented reference data to optimize network parameters. During inference, the trained model generates segmentation predictions on novel images. The model predictions are subsequently analyzed to derive morphometric measurements such as g-ratio, myelin thickness, axon diameter, and eccentricity.

To optimize the quality of the semantic segmentation masks and enhance the robustness of the subsequent analyses, a custom model was trained specifically for this dataset, as detailed in the next section. The model is trained within the nnU-Net framework^24^ and packaged for inference in AxonDeepSeg.^20^ Following segmentation of axonal and myelin structures, AxonDeepSeg automatically computed a comprehensive set of morphometric features for each identified axon. These features include axon diameter, myelin thickness, axon area, and g-ratio. A post-hoc screening procedure was implemented to filter out segmentation artifacts, such as axons with physiologically implausible g-ratios. These curated morphometric data were then utilized for subsequent statistical analyses and inter-group comparisons.

### 2.6 Active Learning Segmentation Strategy

Axon and myelin segmentation was performed using a 2D U-Net architecture^19^ trained via an active learning strategy. This architecture is summarized in Fig. 1(c). This iterative approach enabled efficient model training with a progressively expanding dataset, concurrent with image acquisition, thereby maximizing segmentation accuracy while minimizing manual annotation effort. The model underwent eight iterative training cycles, each utilizing an expanded dataset. Initially, a model was trained using a small, manually annotated dataset comprising three 1000×500 images. For each subsequent iteration, the current model was applied to a new set of images from newly acquired subjects. Table 1 summarizes the content of the data at every training iteration. Predictions selected for correction were chosen based on a qualitative assessment of segmentation quality, prioritizing those with the most apparent inaccuracies. Manual annotation was performed by four individuals with experience in neurohistological image interpretation. To ensure consistency, annotators were explicitly instructed to segment only those structures for which axonal identity could be definitively confirmed, excluding any ambiguous or uncertain cases from the training dataset. Annotators focused on segmenting the myelin rings, and the axon class was obtained by filling the holes in their corrected myelin masks. Only myelinated fibers are imaged because the CARS signal specifically targets the symmetric ${\mathrm{CH}}_{2}$ stretching frequency at $2845\;{\mathrm{cm}}^{-1}$, a frequency prominent in lipids that compose the myelin sheath. As a result, unmyelinated axons are not included in this analysis as they are not visible. These corrected segmentations were then incorporated into the training set, and the model was retrained. Manual mask correction was performed in Gimp, Affinity Designer, and Fiji. All 2D segmentation models were trained with the nnU-Net framework.^24^ The final version of our dataset contained 124 images covering 30 subjects, and here, we applied this final model to six “control” subjects. Our model can be used within the AxonDeepSeg software and is available for download.^25^

**Table 1:** Training iterations.

Training iteration	Number of images	Number of axons	Number of subjects
1	3	368	2
2	12	738	6
3	27	771	6
4	35	1067	8
5	53	1722	11
6	68	2308	15
7	100	3212	22
8	124	3506	30

### 2.7 Model Training and Validation

To reduce overfitting, during the final training iteration, the dataset was split according to a fivefold cross-validation scheme. An ensemble of the five models is used for the final inference on the whole dataset. We report an average validation Dice score of 0.34 (axons) and 0.32 (myelin), with mean IoU values of 0.23 and 0.21, respectively. Although these values are lower than those typically seen for large structures (e.g., tumors or organs), this is a known limitation of overlap-based metrics when applied to small structures approaching the resolution limit.^26^ For myelin sheaths that are often only 2 to 4 pixels thick, a single-pixel displacement in the prediction—which is often within the range of inter-rater variability—drastically penalizes the Dice score, even if the resulting morphometry (diameter/thickness) remains accurate.

To ensure these low overlap scores did not reflect a failure to capture biological reality, the active learning strategy included expert-in-the-loop validation. At each iteration, segmentations were visually inspected, with a priority on correcting morphologically implausible predictions (e.g., broken sheaths, merged axons) over minor pixel-level boundary refinements. This ensured the model converged on biologically meaningful features rather than just maximizing pixel overlap.

The model successfully reproduces established biological relationships, such as the significant positive correlation between axon diameter and myelin thickness (p<0.001), providing evidence for the methodological validity of the segmentation. Furthermore, it is important to view the deep learning model as one component of the full pipeline. As detailed in the next section, the analysis process includes strict filtering for physiological plausibility.

### 2.8 Segmentation Post-Hoc Quality Control

Automated segmentation workflows can generate spurious artifacts that necessitate systematic filtering prior to morphometric analysis. To ensure data quality and remove false positive detections, a multicriteria post-hoc screening procedure was implemented. Axons yielding physiologically implausible g-ratio values outside the range of 0 to 1 were excluded from analysis as such values indicate erroneous segmentation of axonal or myelin boundaries. In addition, axons with diameters below 0.335  μm were systematically removed from the dataset. Given the pixel size of 0.166  μm, axons smaller than 0.335  μm correspond to structures with diameters less than 2 pixels, which are susceptible to inaccurate measurement due to partial volume effects and may represent either segmentation artifacts or genuine axonal structures below the reliable detection threshold of the imaging system. In addition, axons exhibiting eccentricity values exceeding 0.9 were excluded from analysis as the downstream quantification algorithm works under the assumption that axons have a circular shape, which may inadequately represent highly elliptical axonal profiles, potentially introducing systematic measurement errors. This quality control framework ensures that subsequent morphometric analyses are conducted exclusively on accurately segmented axonal structures with reliable geometric measurements.

Morphometric quantification of axon diameters was performed by calculating the diameter of an area-equivalent circle corresponding to each segmented axon. The g-ratio computation involves two diameter estimations: (1) inner diameter calculation utilizing solely pixels designated to the axon class and (2) outer diameter calculation incorporating the aggregate of pixels assigned to both axon and myelin classes. This approach provides standardized measurements of myelination characteristics across the analyzed fiber population.

**Table 2:** Subject information. Data are demonstrated as mean ± standard deviation.

N total	6
***N* male/female**	3/3
**Age**	61.2±17.8
**pH**	6.49±0.34
**PMI (h)**	63.4±20.2

## 3 Results

**Fig. 2:**
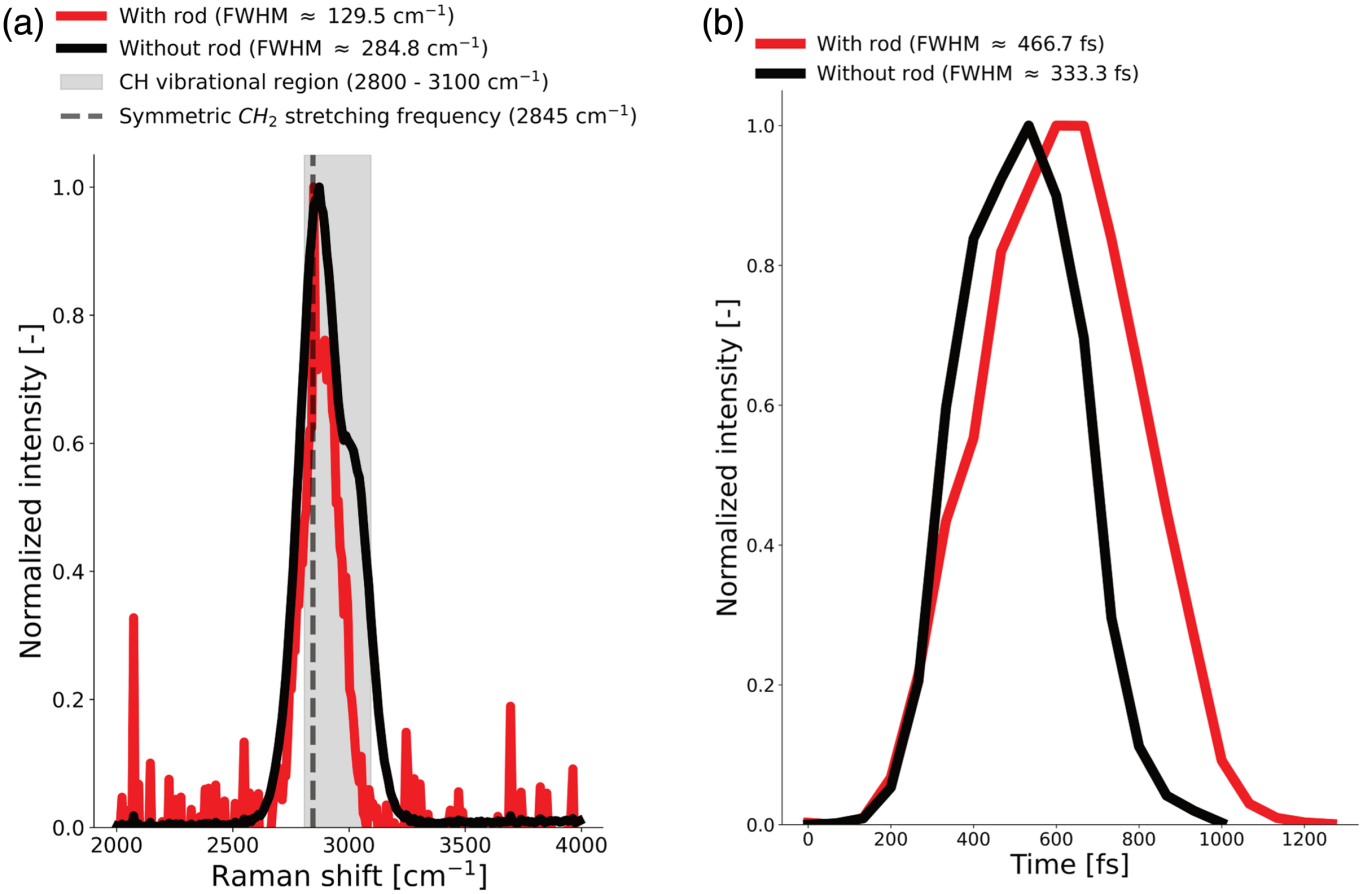
Spectral-focusing CARS (sf-CARS) microscopy has a spatial resolution that is sufficient to target lipids in myelin. (a) The spectral resolution of the sf-CARS is improved by ∼45% with the presence of the glass rod in the excitation path. The detected CARS signal spectra were measured with (red) and without (black) the glass rod. Adding the glass rod reduces the full-width half-maximum (FWHM) of the detected peak from ∼284.8 to $∼129.5\;{\mathrm{cm}}^{-1}$. The detected CARS signal overlaps the CH vibrational region from 2800 to $3100\;{\mathrm{cm}}^{-1}$ (gray area) and the symmetric ${\mathrm{CH}}_{2}$ stretching frequency at $2845\;{\mathrm{cm}}^{-1}$ (dotted vertical line), which are prominent in lipids (14), the main component of myelin. (b) The temporal cross-correlation of the two pulses is measured through the CARS process. Adding a glass rod in the excitation increases by ∼39% the temporal overlap through group velocity dispersion. The intensity of CARS images is measured with (red) and without (black) the glass rod while systematically changing the delay stage position.

The sf-CARS system shows sufficient performance to assess myelin in human brain tissue (Fig. 2). Adding the silica glass rod to the excitation path narrows the spectral linewidth of the CH stretching vibrational region by ∼45% [Fig. 2(a)], showing an increase in spectral resolution. Furthermore, the addition of the glass rod increases the temporal overlap between the pump and the Stokes beam by ∼39%. This change in the cross-correlation of the two stretched pulses is quantified by the mean CARS intensity during sf-CARS imaging of polystyrene beads [Fig. 2(b)].

**Fig. 3:**
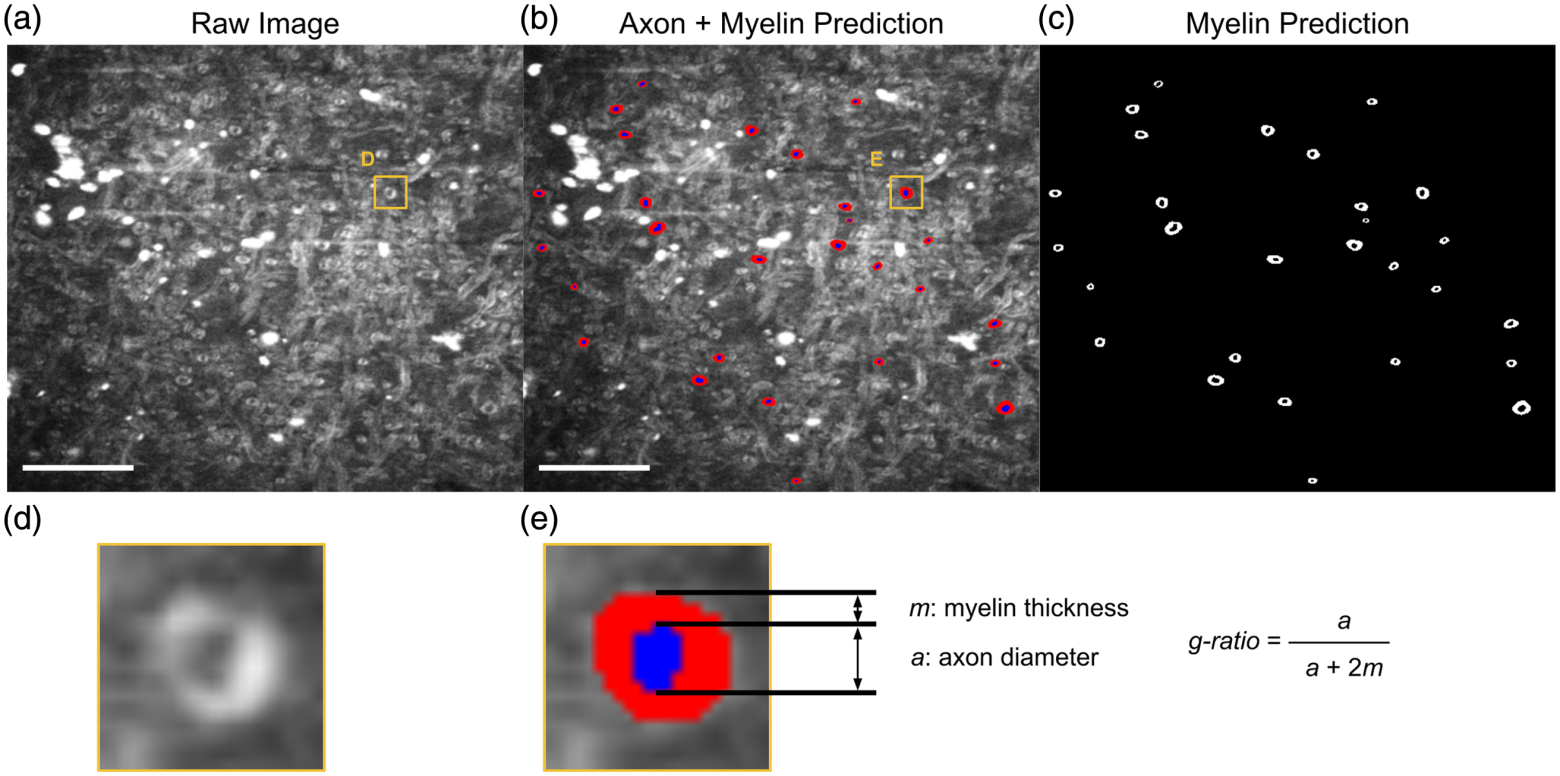
Example segmentation of axons and myelin from an sf-CARS image using a custom-trained AxonDeepSeg deep learning model. (a) Raw, uncorrected sf-CARS image. Bright white spots indicate artifacts of concurrent 2-photon emission from the tunable laser beam. Scale bars = 20  μm. The yellow square highlights the structure of interest [enlarged in panel (d)]. (b) Segmentation of myelin (red) and axon (blue) on top of the raw sf-CARS image. Scale bars = 20  μm. The yellow square highlights the structure of interest [enlarged in panel (e)]. (c) Binary image of myelin segmentation. Scale bars = 20  μm. (d) Zoomed-in structure from raw image (a). (e) Zoomed-in structure from segmented image (b), in which the axon diameter (a) and myelin thickness (m) boundaries are indicated. The corresponding g-ratio formula is provided on the right-hand side.

The subject information for the brain donors is shown in Table 2. After filtering for diameter greater than 0.335 and eccentricity less than 0.9, we were left with 2645 axons. An example segmentation is demonstrated in Fig. 3.

**Fig. 4:**
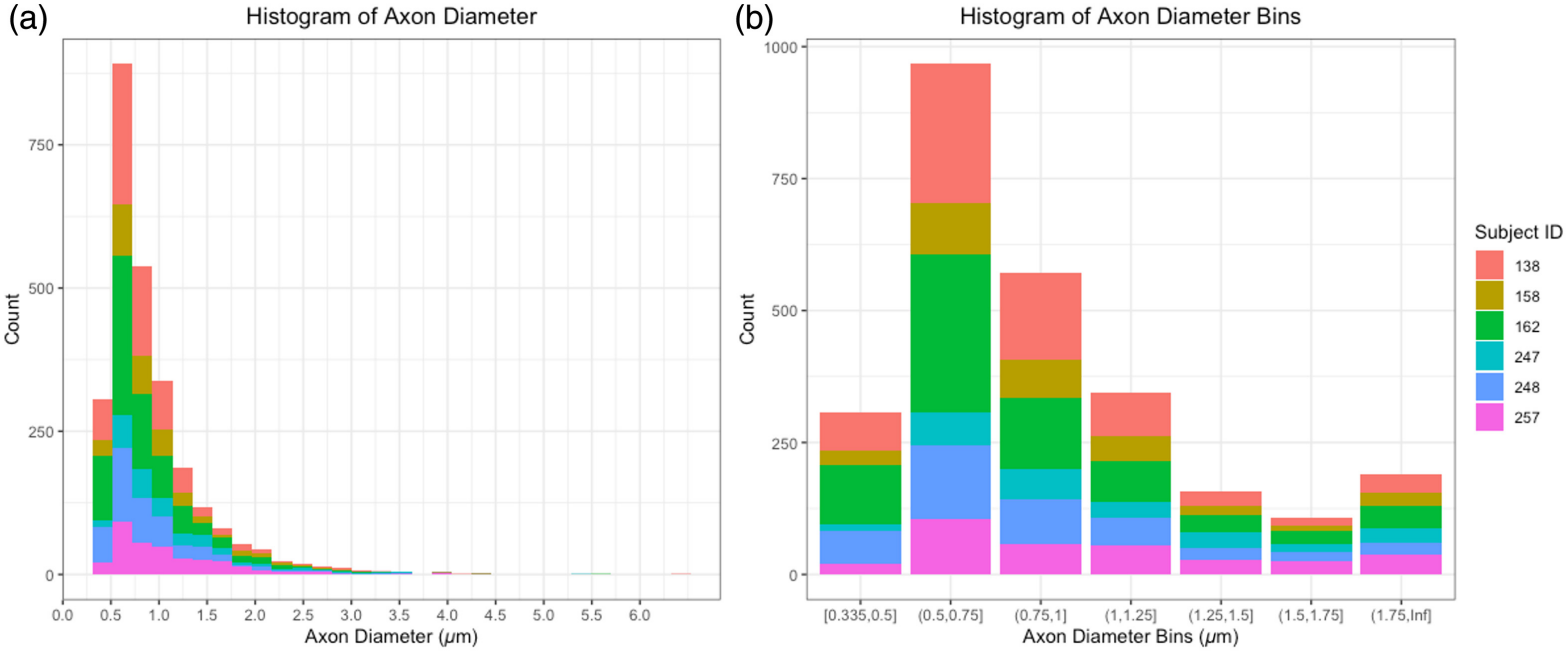
Axon diameter distributions. (a) Histogram of axon diameter distribution, colored by individual subject ID. (b) Histogram of binned axon diameters colored by individual subject ID.

Axon diameters ranged from 0.37 to 6.38  μm, as demonstrated in the histogram in Fig. 4(a). The average axon diameter was 0.93  μm±0.54. We then stratified the axons by diameter bin, starting the cutoff at 0.335 to 0.35  μm, and then increasing by 0.25  μm increments until 1.75  μm. The final bin is 1.75  μm to the maximum diameter (1.75  μm+). The 0.5 to 0.75  μm bin contained the most axons, and the full binned distribution can be observed in Fig. 4(b).

**Fig. 5:**
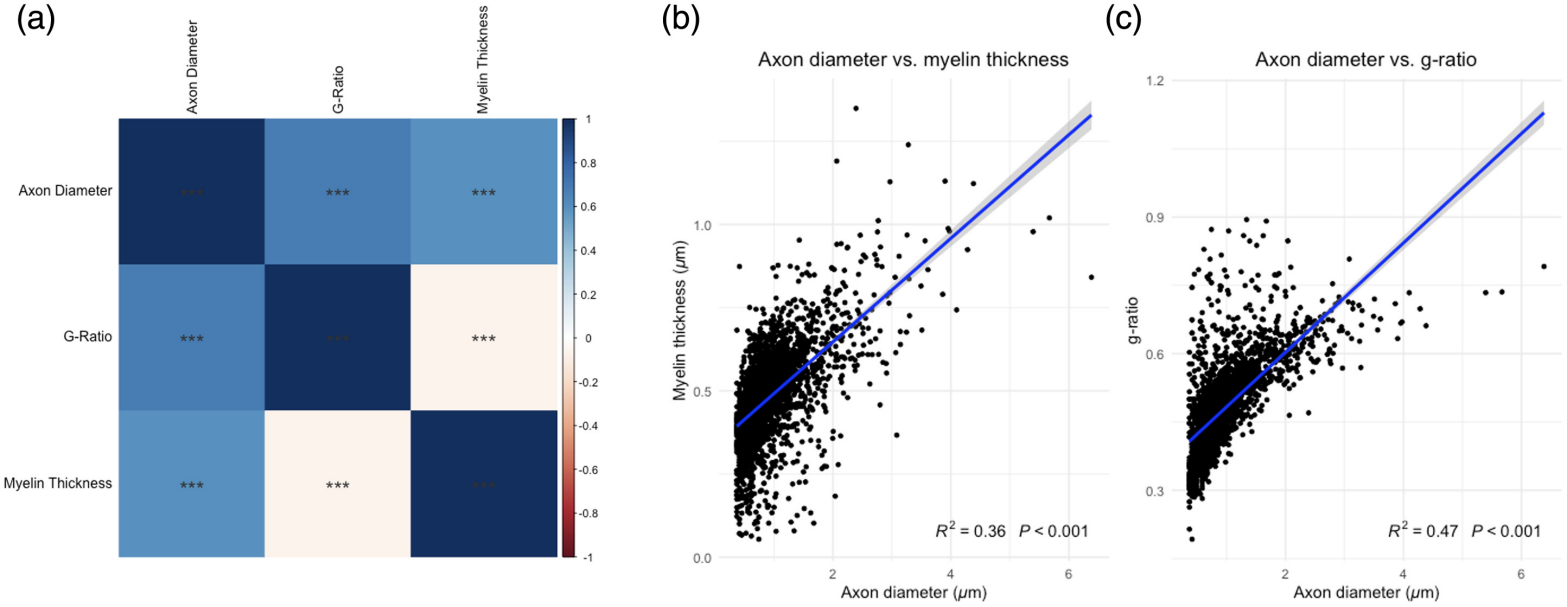
Morphometrics show the expected relationships. (a) Correlation plot showing expected significant correlation across metrics. The color bar represents the correlation coefficient with positive coefficients in blue and negative coefficients in red, and the significance stars represent p<0.01. (b) Scatterplot showing a directly proportional relationship between axon diameter and myelin thickness with trendline in blue ($R^{2}=0.38$, p<0.001). (c) Scatterplot showing a directly proportional relationship between axon diameter and g-ratio with trendline in blue ($R^{2}=0.47$, p<0.001).

**Fig. 6:**
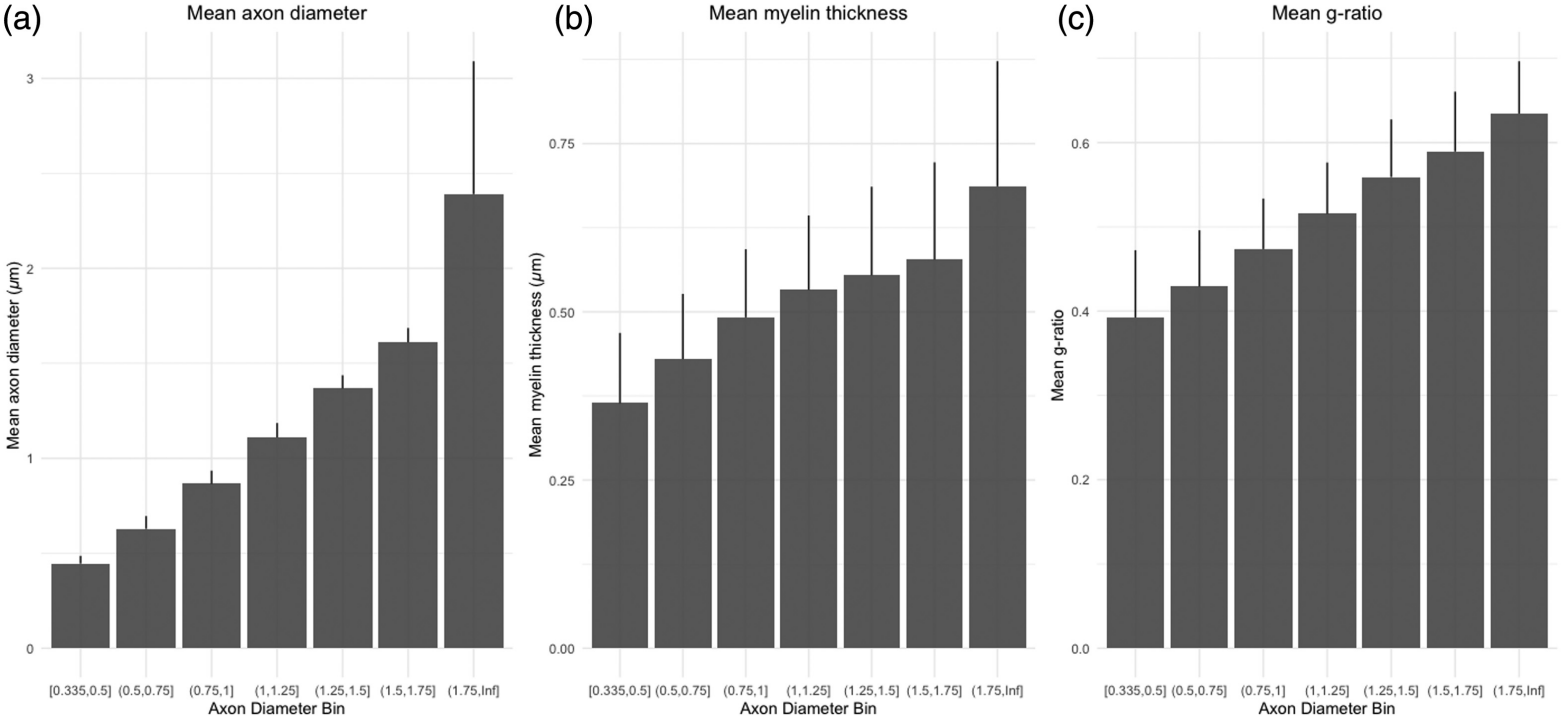
Bar plot demonstrating mean morphometrics across axon diameter bins. Data are shown as mean + standard deviation for (a) axon diameter, (b) myelin thickness, and (c) g-ratio.

Across all fibers, the mean myelin thickness = 0.48  μm±0.14, and the mean g-ratio = 0.47  μm±0.094. The mean myelinated fiber density is $844.36\;\text{axons}/{\mathrm{mm}}^{2}$. We cannot estimate the total axon density (which includes both myelinated and nonmyelinated axons) because the sf-CARS is specifically tuned to map out lipid-rich structures such as myelin. We validated that the morphometrics display the correct relationships with each other, summarized with a correlation plot [Fig. 5(a)], with all correlations showing statistical significance (p<0.001). All correlation coefficients were positive except for the correlation between myelin thickness and g-ratio, which are inversely correlated as expected. We observe that, as expected, myelin thickness increases as a function of axon diameter ($R^{2}=0.38$, p<0.001) [Fig. 4(b)]. Similarly, as expected, g-ratio increases as a function of axon diameter ($R^{2}=0.47$, p<0.001) [Fig. 5(c)]. The mean of each morphometric is summarized across axon diameter bins in Fig. 6.

**Fig. 7:**
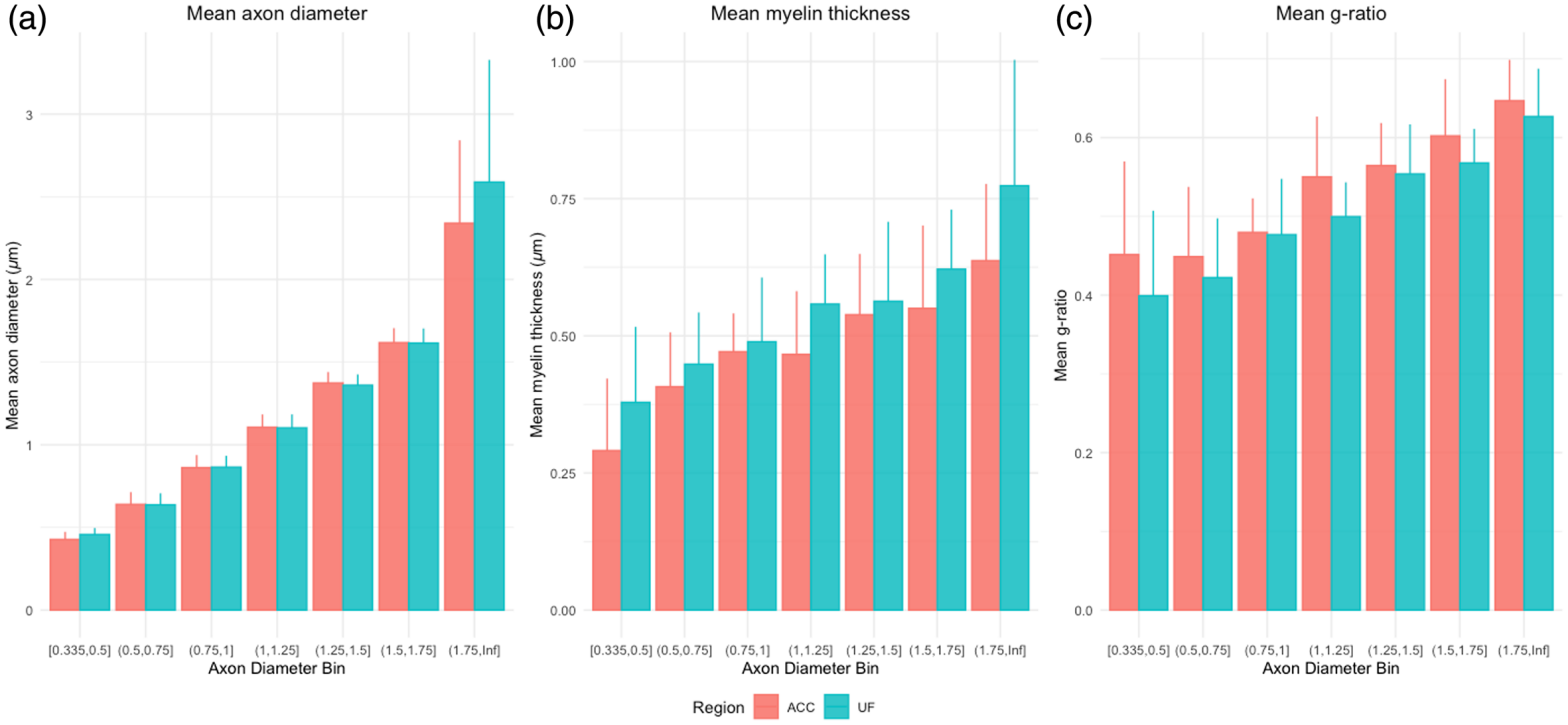
Bar plot demonstrating mean morphometrics for a single subject across regions stratified by axon diameter bins for (a) axon diameter, (b) myelin thickness, and (c) g-ratio. Data for ACC are shown in pink, those for UF are shown in teal.

To further validate the new pipeline, we elected to measure fibers of the ACC WM in a subject for which we also had UF fiber measurements (S247). We selected this region as it was used in Lutz et al.,^15^ in which the previous picosecond-pulsed CARS system was employed. The ACC tissue was processed identically to the UF tissue. As such, we compared ACC fibers (n=206) and UF fibers (n=234) from the same subject and observed that across all diameter bins, myelin was thicker, and g-ratio was smaller in the UF as compared with the ACC (Fig. 7). The axon diameter and myelin thickness between regions appear similar across every diameter bin, except for those 1.75  μm+, in which the mean UF diameter is larger [Fig. 7(a)].

## 4 Discussion

This article presents a full methodological pipeline for the automated ultrastructural analysis of human postmortem white matter. We validate the use of sf-CARS coupled with a custom-trained AxonDeepSeg segmentation model for the quantification of the human postmortem UF. Furthermore, we provide the first-ever characterization of UF ultrastructure, as well as a comparison between the ACC white matter provided by Lutz et al.,^15^ and the UF. As expected, UF axons had thicker myelin than the ACC white matter axons, consistent with what is known about fiber bundles, which necessitate higher conduction velocities.^27^

We also demonstrate that sf-CARS is feasible for use with human postmortem brain tissue. This approach, compared with the traditional picosecond-pulsed CARS systems used by Lutz et al.,^15^ presents both significant advantages and inherent limitations. A primary strength lies in the utilization of more common and affordable Yb laser technology, enhancing accessibility for research laboratories and allowing multimodal imaging in combination with 2-photon excitation fluorescence. The chirping mechanism substantially increases spectral resolution. In the system’s current configuration, the increase in the spectral resolution is modest. Nevertheless, despite the current impossibility to differentiate specifically the symmetric ${\mathrm{CH}}_{2}$ stretch at $2850\;{\mathrm{cm}}^{-1}$, the asymmetric ${\mathrm{CH}}_{2}$ stretch at $2880\;{\mathrm{cm}}^{-1}$ and the ${\mathrm{CH}}_{3}$ stretch at $2930\;{\mathrm{cm}}^{-1}$, the sf-CARS presented in this study shows sufficient performance to image the myelin in biological tissue as all types of CH bonds are naturally present. It would be possible to improve the spectral resolution by introducing a high-dispersive rod if needed in further applications where, for instance, the ${\mathrm{CH}}_{2}$ and ${\mathrm{CH}}_{3}$ lines need to be imaged separately. This approach also introduces the trade-off of dispersing laser power across the entire pulse spectrum, reducing effective power density at specific wavelengths. The current implementation remains suboptimal as images still necessitate post-acquisition contrast correction, highlighting areas for technical refinement. Although traditional picosecond systems may offer greater robustness,^14^ the sf-CARS method promises superior adaptability and multiplicity of information capture. Although hyperspectral imaging was not performed in this study, sf-CARS could enable sweeping of a broader Raman spectrum through larger chirped wavebands. This approach offers more comprehensive and efficient information acquisition compared wth previous systems.^17^^,^^28^ Future developments should focus on optimizing chirping efficiency through higher dispersity materials and exploring three-dimensional acquisition capabilities to enhance visualization of axonal structures and identify structural weaknesses at greater tissue depths. In addition, this approach holds promise for monitoring myelin structural changes through differentiation and intensity analysis of the three distinct lipid peaks in Raman spectra, potentially providing novel insights into myelin pathology and neurodegeneration mechanisms.

Our data processing pipeline was better at detecting small-diameter (<1  μm) axons compared with the tissue imaging and segmentation methods employed by Lutz et al.^15^ As such, it is key for the field to standardize the segmentation methods used, especially when making comparisons across different brain regions. The implementation of deep learning methodology enables unprecedented scalability in morphometric analysis, facilitating the quantification of substantially larger axonal populations than previously feasible with manual approaches.^29^^,^^30^ Although automated segmentation inherently introduces potential sources of variability compared with expert manual annotation, our comprehensive post-hoc quality control protocol aims to mitigate these uncertainties, enhancing the reliability of extracted morphometric parameters across the analyzed dataset. In addition, the current methodology quantifies myelinated fibers within a 2D image plane. Although this approach is sensitive to fiber orientation, which limits the inclusion of fibers that are not roughly perpendicular to the imaging plane, we show that robust WM morphometrics can be extracted across diverse subjects and a wide range of fiber calibers. This demonstrates the method’s ability to characterize heterogeneous fiber populations in different brain areas, such as the ACC and the UF, where myelinated fibers have different directionality properties. To overcome orientation bias, future work could acquire 3D images to map the complexity of fiber bundles, though this necessitates more advanced computational workflows. Finally, a minimum axon diameter threshold of 0.355  μm was applied to ensure measurement reliability. To overcome this spatial resolution limitation, future work should investigate super-resolution approaches such as deconvolution or learning-based retrieval of sub-resolution features. For example, it was shown that the Richardson-Lucy deconvolution method could improve spatial resolution by a factor of at least 2.^31^

There are some noteworthy tissue-specific limitations to this study. The particular part of the UF used in this study (dissected at the temporal pole) represents the terminations of the fiber tract.^32^ In other words, the fibers tend to fan out to reach their targets, so the tract is therefore less densely compacted. As such, the fiber density is probably lower in this subsection compared to much of the UF. As this analysis only considered the terminations of the UF temporal segment, these findings may not hold for the frontal segment or the insular segment.^33^ It would be beneficial for future studies to compare CARS ultrastructure at different segments of the UF to evaluate potential intra-tract variability in ultrastructure.

As previously mentioned, the current sf-CARS configuration does not capture nonmyelinated axons and, as such, likely underestimates the fiber density. Although most white matter tracts are not fully myelinated, we would still expect a majority of UF fibers to be myelinated,^34^ ensuring a representative sampling of the UF. Furthermore, only axons that are roughly perpendicular to the cross-section were effectively measured; thus, directionally incoherent axons were not considered. However, as we cannot confidently attribute noncoherent axons to the UF,^35^ we omitted them from consideration. There are also important UF laterality differences reported,^2^^,^^36^ and it is worth noting that we are only studying the left hemisphere in this study. For example, the left UF has been implicated in select supportive linguistic functions such as proper name retrieval.^37^ On the other hand, psychopathy and acquired criminality studies show a consensus that the right UF shows lower fractional anisotropy values compared with controls.^38^ Other conditions report mixed findings with respect to laterality, such as those related to childhood abuse or socioemotional deprivation.^39^^,^^40^ As such, it would be worthwhile to do a laterality comparison between the left and right UF, ideally at the temporal, insular, and frontal segments. Last, it is important to note that we cannot obtain all ultrastructural measures of interest with CARS, specifically pathology metrics that are only observable at very high resolution such as axonal swelling and myelin splitting.

## 5 Conclusions

In conclusion, we present and validate a full methodological pipeline for the specific detection, imaging, and automated segmentation of myelinated axons throughout postmortem brain sections to obtain quantitative ultrastructural metrics without requiring exogenous markers. We also provide the first-ever descriptions of human UF ultrastructural metrics at the temporal segment. To ensure reproducibility, we supply detailed methodology on tissue fixation and slicing, sf-CARS imaging parameters, as well as model training parameters and access to the trained deep learning model,^25^ which can be used within the existing AxonDeepSeg graphical user interface. We hope that researchers within the postmortem brain field will find this integrated methodology both useful and easily reproducible. Future studies can use this pipeline to interrogate ultrastructural differences in neurological or psychiatric disorders in case-control studies.

## Supplementary Material

10.1117/1.BIOS.3.2.025002.s01

## Data Availability

This model can be used within the AxonDeepSeg software and is available.^25^ The raw microscope images are available upon request.
