# Supplementary material for: Ultrastructural analysis of human uncinate fasciculus with coherent anti-Stokes Raman spectroscopy
Source: Biophotonics Discov. 2026 Mar 17;3(2):025002. doi: 10.1117/1.BIOS.3.2.025002 (PMC13101438; doi:10.1117/1.BIOS.3.2.025002)
Supplement: Supplementary file 1 [file BIOS_003_025002_SD001.pdf]

Ultrastructural Analysis of Human Uncinate Fasciculus with Coherent Anti-Stokes Raman Spectroscopy

Kelly Perlman[1,2*], Valérie Pineau Noël[3*], Armand Collin[4,5*], Justine Major[3], Murielle Mardenli[4], Sébastien Jerczynski[3], Maria Antonietta Davoli[1], Julien Cohen-Adad[4,5], Daniel Côté[3], Naguib Mechawar[1,2,6]

[1] Douglas Mental Health University Institute, Verdun, Canada.

[2] Integrated Program in Neuroscience, McGill University, Montreal, Canada.

[3]CERVO brain research center, Department of Physics, Université Laval, Québec, Canada.

[4] NeuroPoly Lab, Department of Electrical Engineering, Polytechnique Montreal, Canada.

[5] Mila - Quebec Artificial Intelligence Institute, Montreal, Canada.

[6] Department of Psychiatry, McGill University, Montreal, Canada.

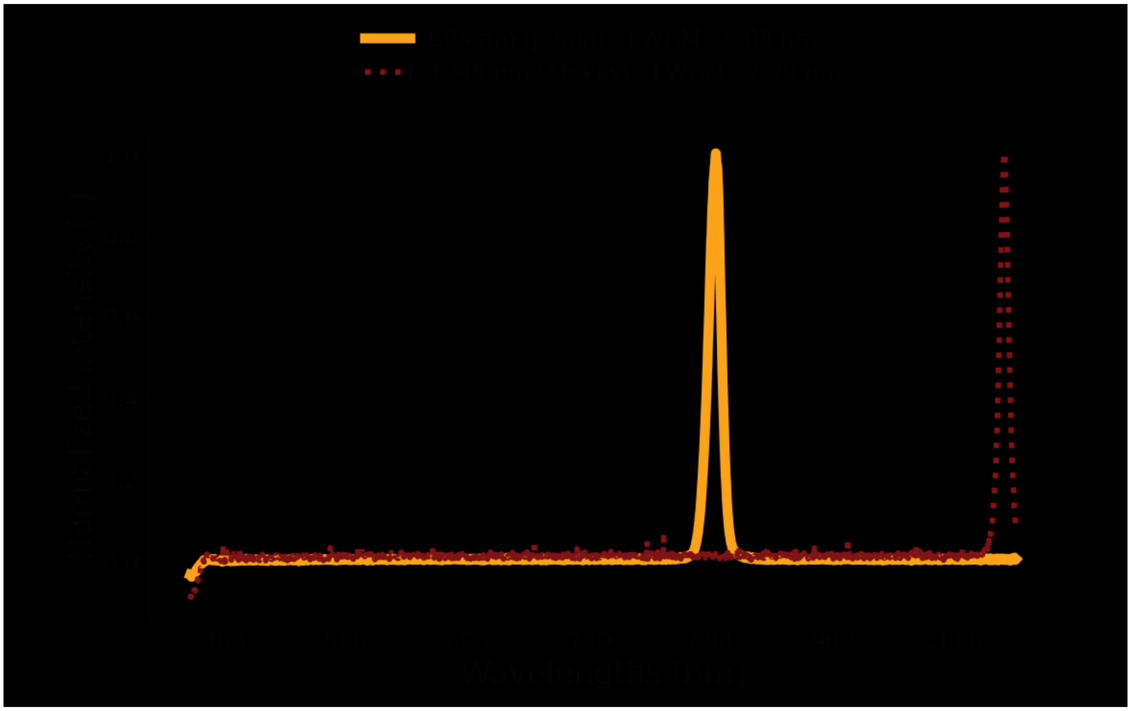

Figure S1 – Spectra of the pump (orange) and the Stokes (botted maroon) beams were acquired before entering the glass rod. The FWHM of the detected signal are 9.08 and 7.29 nm respectively.
